# Implications of gene × environment interactions in post-traumatic stress disorder risk and treatment

**DOI:** 10.1172/JCI185102

**Published:** 2025-03-03

**Authors:** Carina Seah, Anne Elizabeth Sidamon-Eristoff, Laura M. Huckins, Kristen J. Brennand

**Affiliations:** 1 Department of Genetics and Genomics and; 2 Icahn Institute of Genomics and Multiscale Biology, Icahn School of Medicine at Mount Sinai, New York, New York, USA.; 3 Department of Psychiatry, Division of Molecular Psychiatry,; 4 Interdepartmental Neuroscience Program, Wu Tsai Institute, and; 5 MD-PhD Program, Yale University School of Medicine, New Haven, Connecticut, USA.

## Abstract

Exposure to traumatic stress is common in the general population. Variation in the brain’s molecular encoding of stress potentially contributes to the heterogeneous clinical outcomes in response to traumatic experiences. For instance, only a minority of those exposed to trauma will develop post-traumatic stress disorder (PTSD). Risk for PTSD is at least partially heritable, with a growing number of genetic factors identified through GWAS. A major limitation of genetic studies is that they capture only the genetic component of risk, whereas PTSD by definition requires an environmental traumatic exposure. Furthermore, the extent, timing, and type of trauma affects susceptibility. Here, we discuss the molecular mechanisms of PTSD risk together with gene × environment interactions, with a focus on how either might inform genetic screening for individuals at high risk for disease, reveal biological mechanisms that might one day yield novel therapeutics, and impact best clinical practices even today. To close, we discuss the interaction of trauma with sex, gender, and race, with a focus on the implications for treatment. Altogether, we suggest that predicting, preventing, and treating PTSD will require integrating both genotypic and environmental information.

## Introduction

Psychiatric disorders are characterized by marked clinical heterogeneity in presentation, trajectory, and treatment responsivity, which likely reflects the interactions of myriad genetic and environmental risk factors. Among the most significant environmental risk factors is traumatic stress, increasing risk of major depressive disorder (MDD) (1), bipolar disorder (2) schizophrenia, eating disorders, post-traumatic stress disorder (PTSD) (3), and many other disorders (4–7). The joint role of stress and genetics have particularly been studied in PTSD (8–13), highlighting inherent mechanisms of stress susceptibility.

The scale of varied individual genetic and stress exposure profiles suggests that there are many mechanisms of risk that converge on disorder manifestation. Therefore, it is unlikely that a single therapeutic will suffice to benefit all patients with a disorder. Instead, precision approaches to therapeutics, tailored to individual mechanisms of risk, are likely necessary to address the biological basis of psychiatric disorders. Precision medicine is the concept of taking into account individual genetic, molecular, and environmental profiles to target therapeutics to individualized profiles of risk (14). To achieve this, these risk elements must be elucidated fully to differentiate the most effective therapeutic options for each profile.

**Figure 1:**
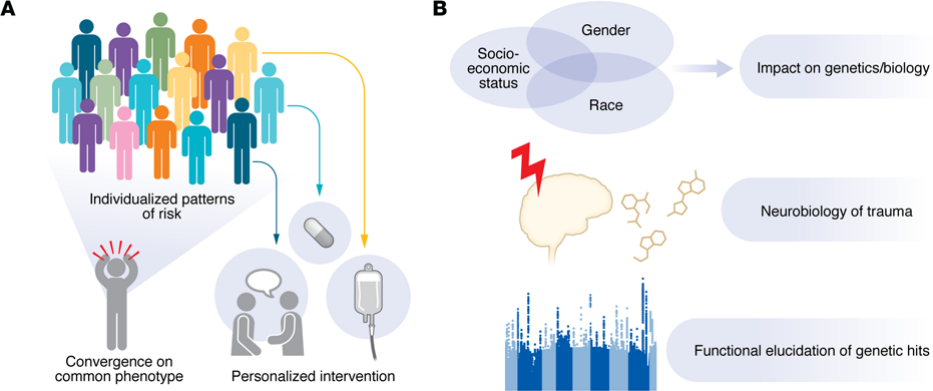
Schematic of scientific rationale and methods to understanding gene × environment interactions in post-traumatic stress disorder. (**A**) Post-traumatic stress disorder (PTSD) is a heterogeneous disorder characterized by individualized patterns of risk requiring individualized interventions, despite convergence on a common phenotype. (**B**) PTSD is the result of both genetic and environmental risk factors as well as the impact of trauma. The neurobiology of each of these risk factors should be characterized independently and jointly in order to advance our understanding of PTSD pathophysiology. This includes understanding the biological impact of the underlying socioeconomic and home environment, delineating the long-term encoding of traumatic exposures on the brain, and identifying how genetic risk functionally affects brain neurobiology. The functional elucidation of genetic hits will require linking genetics to downstream-omics, such as transcriptomics and epigenetics, exploring genetic models of disorder risk, genetics-matched electronic health records, and clinical and imaging data.

Here, we review current understandings of the genomics of stress disorders, with an emphasis on how large-scale genetic studies may be integrated with clinical and epidemiological data outlining the role of environmental stress to elucidate the functional mechanisms underlying individualized risk. In particular, we use PTSD to showcase the technological advancements that have recently enhanced psychiatric genetics research. We discuss how these tools lead to an integrated understanding of gene × environment (G×E) interactions that may be leveraged to generate clinically actionable information and advance precision psychiatry for the holistic, individualized care of all patients (Figure 1).

## Advances in PTSD genetics

PTSD is heritable, with twin studies estimating the genetic component of heritability to be around 30% (15) and SNP-based heritability estimated from GWAS ranging from 5% to 20% (16–19). This heritability differs according to sex (20) and trauma type (21, 22). Early biological theories of genetic risk for PTSD centered around hypothalamic-pituitary-adrenal axis dysregulation (23, 24) and*,* in particular, alterations in glucocorticoid receptor sensitivity (25). Today, GWAS facilitate hypothesis-free genetic discovery. Major insights from GWAS include identification of hundreds of genomic loci associated with psychiatric disorders (26–29); elucidation of the polygenic architecture of psychiatric traits and disorders, where many variants with small effect sizes confer additive disorder risk; and the localization of a majority of disorder-associated variants in noncoding regions of the genome.

The most recent GWAS of PTSD (19) examined 1,222,882 individuals (137,136 cases), identifying 95 genome-wide significant loci and prioritizing GABAergic cell types as particularly enriched associated with PTSD risk. Previous GWAS conducted in the Million Veterans Program cohort (17, 18) assessed the genetic contribution to quantitative PTSD symptom domains (e.g., reexperiencing, hyperarousal, avoidance, and a total symptom severity index), demonstrating that distinct genetic risk factors confer risk for particular symptoms, lending credence to the notion of biologically derived “endophenotypes.” Altogether, the stated goal of large-scale genetic studies has been to facilitate early screening and intervention for vulnerable individuals, inform the biological mechanisms of PTSD, and identify potential avenues for therapeutic interrogation.

### Genetic screening for individuals at risk of PTSD.

The clinical hope of GWAS has been to better quantify genetic risk of disorders. To derive a measure of risk for highly polygenic traits, polygenic risk scores (PRS) (30) aggregate the trait-associated effect sizes of trait-associated SNPs to provide individual estimates of SNP-derived risk. In PTSD, individuals in the highest quintile of risk have 2.8 times the odds of developing the disorder, a value far from deterministic and unlikely to merit true clinical stratification. Yet, to date PRS have shown greater promise outside of psychiatry, with the capacity to distinguish individuals at high risk for cardiovascular disease (31), breast cancer (32), and familial hypercholesterolemia (33) and even predict treatment response (34). Within psychiatry, the PRS for schizophrenia currently has the highest predictive potential (35), albeit below that of cardiovascular disease, explaining only 7% of disorder variance (36). PTSD PRS explains 6.6% of disorder phenotypic variation (19). This may reflect the more complex environmental contributions to psychiatric disorders, particularly PTSD, or merely sample size and phenotype definition constraints in psychiatric GWAS.

Attempts to use PRS to predict PTSD among subpopulations of individuals exposed to specific traumas have been mixed. Neither a diagnosis of PTSD nor any symptom dimensions were predicted by a PTSD-specific PRS among 9/11 responders (37). However, a PTSD PRS was significantly associated with the presence of PTSD 6 months following mild traumatic brain injury and explained 7% of disorder variation (38). Among US army soldiers deployed to Afghanistan, a PTSD PRS was significantly associated with greater severity of PTSD symptoms, though with a low effect size (39).

Approaches to calculate PRS are still evolving, with newer methods able to account elegantly for complex relationships between variants, and incorporate trans-ancestry prediction (40–42); however, predictive potential still varies greatly by ancestry. Moreover, assumptions inherent in PRS computation limit the current clinical utility of PRS. Risk variants are weighted by their effect size and combined without consideration of interactions of risk variants within biological pathways, cell types, or developmental contexts. Additionally, PRS does not consider environmental information, though we know G×E interactions are critical in disorder risk and progression (see below). Given the low positive predictive value of current measures, it is difficult to determine the appropriate role of PRS in psychiatry at the level of the individual patient.

With substantial improvements in incorporating environmental measures of risk, as well as enhancement of cross-ancestry portability, it is possible that PRS could be used to identify patients at risk for PTSD and other psychiatric disorders and institute earlier screening than the physician otherwise would. In this way, PRS has potential to be another decision-making tool to discern pretest probability when making diagnoses.

### Genetically driven insights into the biological mechanisms of PTSD.

GWAS provide a means for genetic discovery on an unprecedented genome-wide scale but are limited in a number of ways. First, the coinheritance of risk variants due to linkage disequilibrium makes it challenging to resolve which variant is causal for the trait and which others are merely correlated. Furthermore, the majority of SNPs identified by GWAS are located in noncoding regions of the genome and so must be integrated with other functional data (43), such as epigenetic, transcriptomic, and proteomic data, to identify putative causal mechanisms (e.g., as promoters or enhancers) associated with variant-associated risk. By integrating genetic data from PTSD cases and controls with transcriptomic and epigenomic data from postmortem brain or cultured human neurons and glia, it is possible to map risk loci with the putative target genes whose expression they regulate.

SNPs that regulate the expression of nearby genes are termed *cis-*expression quantitative trait loci (*cis-*eQTLs), whereas those that modify the expression of distant genes are termed *trans*-expression quantitative trait loci (*trans*-eQTLs). eQTLs are highly enriched for disease heritability (44) and conserved across ancestries (45). Genetic variants (dys)regulate biology beyond expression, associating variants (xQTLs) with changes in methylation (46), chromatin accessibility (47), splicing (48), and allele-specific expression (49), all converging on the regulatory mechanisms impacted by disorder-associated variants. Integrating eQTLs with GWAS loci may take the form of colocalization analyses, comparing genetic architecture of GWAS and xQTL loci, or transcriptomic imputation (TI). TI approaches leverage eQTL relationships to predict tissue- or cell-type-specific gene expression from genotype, effectively translating GWAS associations to tissue- and cell-specific gene associations. TI studies have identified genes dysregulated by GWAS-associated variants across schizophrenia (50), bipolar disorder (51), anorexia nervosa (52), PTSD (22), and cannabis use disorder (27).

In addition to modifying methylation (13), open chromatin (53–55), and transcription factor binding sites (56), GWAS-identified SNPs may biologically confer risk via long-range regulatory mechanisms (57). To profile such mechanisms, GWAS can be mapped to distal targets via interchromosomal interactions (58), such as chromatin loops (59). Studies of the three-dimensional genome thus identify the changing chromosomal landscape in cell-type-specific (60), developmental stage–specific, and disorder-specific contexts (61), which causally regulate the activity of risk alleles through long-range regulatory effects (60).

While these genomic approaches can identify putative links between risk variants and predict gene targets, experimental strategies are required to definitively identify the causality of GWAS-associated variants. Targeted strategies such as CRISPR base editing (62) and prime editing (63) allow for the precise editing of single variants, allowing for insight into their downstream ramifications. However, these strategies are experimentally difficult to conduct at scale and require high confidence that a variant edited be causally implicated in disorder phenotypes. More scalable strategies include CRISPR inactivation (CRISPRi) (64) or activation (CRISPRa) (65), which is achieved by fusion of a catalytically inactive Cas9 protein to transcriptional repressors or activators. CRISPRi and CRISPRa have been used for pooled CRISPR screening, a technique where these repressors and activators are targeted to a number of sites and resolved by single-cell sequencing (66, 67). This has allowed for the parallel investigation of hundreds of targets and their downstream disorder-associated pathways (68–70). Beyond this, massively parallel reporter assays (MPRAs) may be used to validate GWAS-associated variants at an unprecedented scale (71). MPRAs introduce thousands of candidate variants by pairing barcoded variants with fluorescently tagged reporters behind a minimal promoter in a plasmid and delivering these into a cell type of interest. Then, the relative gene expression of the reporter gene is assessed in comparison to the allele present, allowing for a measure of whether the allele has transcriptional activity (72). MPRAs have successfully demonstrated that hundreds of GWAS SNPs regulate gene expression (73), validating the functional role for variants involved in MDD (74) and schizophrenia (75). However, MPRAs do not identify whether, in their endogenous context, alleles impact specific downstream targets, as expression of a reporter is being assessed.

With the discovery of new PTSD-associated loci in the most recent GWAS, MPRAs may be used to interrogate each of the 95 PTSD-associated loci to assess which of the many variants in high linkage disequilibrium causally impact gene expression. This would allow for a functional understanding of the causal variant and perhaps improve specificity to clinical uses of the GWAS, such as PRS.

### Novel genetically informed therapeutics.

To design pharmacological therapies for PTSD, disorder-implicated biological pathways must first be identified before they can be targeted. Thus, knowing the mechanism by which a particular GWAS variant in a noncoding region regulates a gene target and its associated pathway is crucial information, beyond whether or not that variant is associated with disorder risk. Complicating this, the biological pathways affected by genetic variants may change under varying biological contexts. For instance, studies on the developing and adult brain indicate that genetic regulation is timing dependent, suggesting that pharmacological or other therapeutic interventions at specific time points may have outsized impact on the onset or progression of a disorder (76). This is consistent with developmentally critical periods, particularly those hypothesized to affect PTSD via windows of childhood trauma susceptibility (77).

A strategy toward development of genetically informed therapeutics is combining GWAS with electronic health record (EHR) data. EHR data standardizes encoding of diagnostic and therapeutic information and allows for easy mining of longitudinal health information across individuals’ lifetimes. Particularly with advances in natural language processing models (78), clinical vignettes may now be systematically parsed and quantitatively coded (79). This allows for better insight into comorbidity patterns and clinical profiles associated with particular genotypes, which may be used to further diagnostic profiles for precision medicine.

Linking EHR-derived profiles to genetics can be accomplished via phenome-wide associated studies (PheWAS) (80, 81) in EHR-matched biobanks. These studies probe the potential clinical consequences of a genetic variant (82) by identifying the diagnostic comorbidities associated with risk variants across individuals’ lifetime diagnostic histories (83); moreover, PheWAS approaches allow integration of physiological and environmental context, beyond the case-control design of GWAS (84). For example, a recent PheWAS of PTSD identified sex differences in PTSD-associated comorbidities, finding a stronger association with osteoporosis and cardiovascular disease in male cases but increased infections and obesity in female cases (83). This finding informs the personalized manner in which secondary screening, counseling, and preventative medicine might be approached by sex in individuals with PTSD.

Beyond diagnostic profiles, genetic data may also be mapped to lab values and imaging results. Similar to PheWAS, LabWAS studies map variation in clinical laboratory results to genetic variants, identifying lab result profiles associated with risk variants (85, 86). LabWAS for PTSD have identified variation in mean corpuscular hemoglobin concentration, neutrophil and eosinophil count, and tau protein as associated with PTSD risk variants (87). For brain imaging data, association with disorder-associated biology has been approached using BrainXcan, a machine-learning algorithm trained on matched genetic and brain MRI data (88). When applied to genetic data, it imputes MRI features associated with that genetic background. Comparison of imputed MRI features between cases and controls may identify suggested diagnostic biomarkers.

Genetic data may also aid in drug repurposing for psychiatric disorders. For example, recent work integrating genetic, transcriptomic, and proteomic data has identified antihypertensive drugs that interact with transcription of the *CACNA1D* gene, which encodes a voltage-gated calcium channel and is associated with psychiatric disorder risk (89, 90). Given the high degree of shared symptoms and genetic risk loci, as well as comorbidity across psychiatric disorders, redeploying pharmacological treatments may enable clinicians to treat in a more individualized manner.

Altogether, realizing the clinical applicability of genetic studies relies on large-scale resources incorporating both clinical and genetic data. Biomedical research initiatives that seek to build a large, diversely populated database such as the NIH All of Us Research Program (91) hold promise for disentangling these variables.

## G×E interactions in PTSD

A major shortcoming of genetic studies in PTSD and potential explanation for the missing heritability in GWAS and low predictive power of PRS is the lack of consideration of environmental risk. Environmental contexts, including housing stability, socioeconomic status, and substance use, contribute heavily to disorder risk or resilience (92, 93). In PTSD, arguably no environmental context is more important than that of traumatic stress, given that trauma exposure is required for the diagnosis (94). The traumatic events named in Criterion A of the Diagnostic and Statistical Manual of Mental Disorders-5 (DSM-5) include exposure to war, physical or sexual trauma, and natural or human-made disasters. Sexual violence especially leads to higher rates of PTSD compared with other traumas (21). The timing, extent, and type of stressor have also shown to play a role in disorder development; for example, childhood trauma, while a third as prevalent as being in an accident, is more than twice as likely to lead to PTSD later in life (95). Experiencing multiple traumatic events also confers increased risk of PTSD, with higher trauma burden linked to higher rates of PTSD (96). Therefore, understanding the biology of trauma may provide insights into the functional mechanisms of PTSD genetic risk.

One manner by which traumatic stress is physiologically mediated is via glucocorticoid signaling through the hypothalamic-pituitary-adrenal axis, where glucocorticoid end-effectors impart lasting changes within the brain (97). Adaptation to stress is also encoded biologically. Differential individual reactions to stress have been observed in rodents, such as glutamate receptor upregulation in the prefrontal cortex mediating resilience to stress, and glutamate hyperactivity in the nucleus accumbens (98).

Understanding the mechanisms by which innate genetics may molecularly mediate responses to the environment is important for developing targeted therapies for individuals with heterogenous genetic and environmental risk burdens. Basic science research in molecular mechanisms and brain circuits has demonstrated that biological vulnerability is manifold, elucidating patterns of risk undetectable by considering genetics or traumatic stress alone.

### Molecular mechanisms underlying stress response.

Stress itself is known to involve multiple genes and biological systems, imparting lasting changes on genetic regulation. Methylation changes associated with early life traumatic stress have been shown to potentially moderate the effects of *FKBP5* genotype, encoding a major moderator of glucocorticoid sensitivity (99). Similarly, a variant, rs2267735, which disrupts a putative estrogen receptor binding site in the *ADCYAP1R1* gene, have been shown to confer a genotype by childhood maltreatment interaction on PTSD incidence and severity only in adult female participants (100). Expression and methylation changes of this gene in peripheral blood have been associated with PTSD (101). rs893290, a variant within the *RORA* gene identified by the first PTSD GWAS, have been shown to interact with childhood physical abuse to influence trajectories of post-traumatic stress symptomology (102). Additional candidate genes identified to interact with environmental risk include *GABRA2* and child abuse (8), *APOE* and combat exposure (9), *ADRB2* and childhood trauma (11), *CNR1* and child abuse (103), and *COMT,* a key player in catecholamine catabolism, where traumatic load modified PTSD susceptibility differentially in individuals with *COMT* polymorphisms (104).

Unfortunately, candidate gene studies often contain selection biases and often analyze homogeneous and small cohorts. Expansion of candidate gene studies to unbiased discovery on a genome-wide scale may be conducted using gene-environment-wide interaction studies (105). Such studies require large sample sizes and are often confounded by the complex intersecting social structures underlying exposure risk (106). As such, they have yet to be completed for PTSD. In MDD, marginal interactions with stressful life events have been identified in relatively homogenous populations (107). In suicidality, traumatic experience and post-traumatic stress as environmental exposures interact with genetic variants underlying suicidality (108). Most likely, the power required to identify a number of interactions on this scale has yet to be reached, but as biobank participation increases, this may soon be feasible.

Whether genetic or environmental, biological consequences of PTSD can be reflected in tissue-specific gene expression. Expression studies of PTSD have largely been conducted on the brain and blood tissues of individuals with PTSD. In the postmortem brain, expression signatures associated with PTSD are largely region specific across the dorsolateral prefrontal cortex, orbitofrontal cortex, and dorsal anterior cingulate cortex. In one study, differentially upregulated genes were enriched in axonal and synaptic transmission while downregulated genes were associated with glial activity (109). Another study identified genes enriched for immune signaling and learning and memory consolidation (110). In peripheral blood, inflammatory and metabolic pathways are largely altered (111). In a quantitative study of PTSD severity, PTSD symptom subdomains were associated with distinct expression profiles in peripheral blood, indicating that particular symptoms, such as anxious arousal, determine distinct transcriptomic profiles (112). Transcriptomic profiling of the dorsolateral prefrontal cortex in PTSD has identified downregulation of genes associated with synaptic signaling (e.g., *ELFN1*) and GABAergic neurotransmission (e.g., *SST*, *GAD2*, *SLC32A1*) (109). Overall, postmortem studies are limited by tissue availability and the impacts of an amalgamation of exposures across the lifespan. As such, it is difficult to disentangle the specific effects of certain exposures on the brain in a controlled manner. Furthermore, it is impossible to conduct causal studies in the postmortem brain. Therefore, to causally understand the molecular pathophysiology induced by both genetics and environmental exposures in PTSD, many groups have utilized various strategies for in vitro and in vivo modeling.

One major mechanism by which environmental exposures moderate genetic regulation is epigenetic modifications (113). Epigenetic modifications can be environmentally induced and are long lasting, potentially even transmitting intergenerational information as a result of environmental exposures (114). Many such epigenetic modifications have been reported as a result of trauma. For example, decreased methylation at the *SLC6A4* locus, a longtime candidate gene for PTSD, has been associated with increased susceptibility of traumatic experiences to induce PTSD (115). Similarly, trauma-dependent demethylation of CpG sites in *FKBP5* (99) reinforced the role of *FKBP5* in PTSD. Epigenome-wide association studies (EWAS) of PTSD (116–119) have aimed to dissect such molecular modifiers associated with PTSD on a genome-wide scale. CpG sites associated with PTSD from EWAS include those associated with immune and inflammatory signaling, transcriptional regulation, axonal guidance, and protein binding.

A major limitation in studying psychiatric disorders is the inability to access neuronal tissue from patients. Human induced pluripotent stem cell (hiPSC) technology has allowed for the generation of neurons in vitro from blood or skin biopsies obtained from patients. Cells are reprogrammed through the hiPSC state and then differentiated into various brain cell types, including neural progenitors (120), astrocytes (121), microglia (122), oligodendrocytes (123), endothelial cells (124), and glutamatergic (125), GABAergic (126), dopaminergic (127), and serotonergic (128) neurons. Reprogramming conserves the individual genetic elements from each donor, allowing insight into the neuronal phenotypes induced by donor-specific genetic backgrounds. hiPSC-derived brain cell types from individuals with various psychiatric disorders have been developed and have allowed for unique insights into the neuronal mechanisms underlying schizophrenia (129), bipolar disorder (130), MDD (131), and substance use disorders (132). Our group similarly derived hiPSC-derived neurons from individuals with and without PTSD and observed that cells from individuals with PTSD demonstrated transcriptional hyperresponsivity to acute exposure to glucocorticoids compared with controls (133). This in vitro causal G×E study demonstrated the utility of hiPSC models in providing a platform for well-controlled, isogenic experiments with equal exposure to researcher-determined stressors.

Current in vitro approaches to examine the genetic interaction with stress exposure have solely focused on the impact of acute glucocorticoid-mediated stress. This is likely an oversimplification of the complex response to stress, which involves many neurotransmitters, hormones, cytokines, and circuits. Expanding the modalities of stress delivered to hiPSC-derived neurons will likely improve our understanding of stress response. Catecholamines, including adrenergic signaling molecules, are an especially relevant next target owing to their role in sympathetic sensitivity (134), which is characteristic of PTSD. Beyond neurotransmitters, the physiological response to stress involves inflammatory mediators and cytokines, such as IL-6, TNF-α, and IL-1 (135). Heightened IL-6, in particular, has been associated with childhood maltreatment (136). Glucocorticoids jointly induce pro- and antiinflammatory mechanisms that have been postulated to represent priming of the immune system to a stressor and restoration of homeostasis after stressor exposure (137).

The timing and chronicity of exposure to stress should also be assessed in future modeling of in vitro stress response. A single glucocorticoid stressor does not capture the chronicity of long-term stress exposure or extended reaction to a stressor. Studies of chronic glucocorticoid-mediated stress in hiPSC-derived astrocytes from patients with MDD have identified gene expression changes unique to long-term stressors compared with acute stress (138). It is unknown how this may impact neurons. Additionally, with the observation that basal peripheral cortisol levels are decreased in individuals with PTSD (139), it is unclear whether chronic glucocorticoid exposure may impart different physiological impacts from an acute stressor. Another avenue of interest is repeated acute stressors, which may allow for assessment of how repeated traumatic events imbue either additive or synergistic effects on the brain.

Evidence assessing the epigenetic landscapes upon reprogramming cells to pluripotency have found that the majority of these signatures are reversed during reprogramming (140). Therefore, it is unlikely cells retain the epigenetic features of traumatic exposure that may be present in donor samples collected from fibroblasts. Therefore, stress exposures in these neurons likely mimic either fetal stress or early life stress, rather than trauma experienced in adulthood. This can be an advantage; impacts of maternal stressors transmitted during pregnancy may be modeled in hiPSC-derived models (141). Exposures, such as neonatal glucocorticoid exposure (142), cytokine exposure (i.e., IL-6) (143), and drug exposures (144, 145) (i.e., THC, nicotine, alcohol), which have been shown to play a role in neurological development and risk for neuropsychiatric disorders, may easily be modeled in this manner. However, while hiPSC-derived models allow for direct experimentation on human neurons, these cells do not demonstrate the complex phenotypes associated with psychiatric disorders.

### Developmental contexts and neurocircuitry in stress response models.

Rodent models of psychiatric disorders provide opportunity for more externally valid stress paradigms (146) such as social defeat stress to induce a more natural physiologic response to stress. Stress paradigms in rodent models recapitulate many aspects of stress, from social threats to life-threatening exposures that cortisol exposure to neurons in a dish inherently cannot. Rodent stress models reveal that stress modifies the brain in an age-dependent and stressor-dependent manner (147). Acute stress, for instance, induces a reduction of hippocampal connectivity to the thalamus but increases connectivity to the amygdala (148). Chronic stress, on the other hand, incites neural remodeling of the medial prefrontal cortex (149). Furthermore, the ability to recover from stressors decreases with age (98). Yet, poststress recovery still leaves a lasting effect on brain structures. In the medial prefrontal cortex, dendritic shrinkage is observed, whereas hypertrophy is observed in the amygdala (150). Rodent studies additionally allow for circuit-level phenotyping at high resolution (146). Optogenetic models (151), for instance, allow for induction of circuits hypothesized to play a role in stress. Retrograde viral tracing (152) additionally allows for mapping of complex circuitry. They allow for examination of developmentally critical periods of stress exposure that lead to long-term encoding of stress response. Rodent models also allow for investigating recovery from stressors, implicating various circuits and genes in vulnerability as opposed to resilience after stress.

### Incorporating implications of G×E interactions in clinical practice.

Interventions for PTSD are still largely broad-spectrum, despite vast clinical heterogeneity in PTSD presentation and treatment response (153). While developing novel genetic approaches to identify therapeutics, it is important to recognize that the many identified mechanisms of G×E interactions suggest heterogeneous etiologies of risk. It is therefore likely that risk for PTSD does not converge upon a common pathway. Acknowledgment that each individual’s genetic makeup as well as their diverse experiences will contribute to their symptomology and the care they will require is important for clinicians to bear in mind.

Beyond PTSD, it is becoming evident that traumatic exposures impart risk for many medical conditions (154–157). As such, in the clinic, collecting a trauma history even when not obviously indicated may elucidate risk for important medical comorbidities. Structured interviewing to identify traumatic experiences across the lifespan (158) should be built into clinical questioning and reasoning around neuropsychiatric disorders.

As traumatic exposures significantly alter neuropsychiatric morbidity (159, 160), it is imperative to advocate for interventions that reduce trauma burden. Primary interventions that have shown to prevent traumatic exposures include educating individuals about healthy and unhealthy relationships and early signs of abuse (161). On a sociopolitical level, advocating for restriction of possession of firearms can reduce community gun violence and exposure to traumatic assault and violence (162). Regarding childhood trauma in particular, it will be important to identify sensitive periods (95, 163, 164) at which children are most vulnerable to traumatic exposures to concentrate resources for primary prevention within these developmental windows.

Important secondary interventions include expanded access to mental health care for those who have experienced trauma, including reducing the financial burden of seeking care. Screening for traumatic interpersonal experiences in primary hospital settings and schools is important to expeditiously identify and prevent domestic and sexual violence (165, 166). Availability of shelters and social workers who can facilitate the safety of individuals at risk for violence can drastically reduce the changes of traumatic violence.

## Intersection of trauma with sex, gender, and race

Rates of PTSD following trauma differ by gender, with two to three times as many women impacted as men (167). Gendered experiences of trauma likely contribute to this disparity (168). Men are more likely to experience accidents, physical assault, and combat trauma, while women are more likely to experience childhood traumas and interpersonal traumas (169), such as rape and sexual assault, which are known to lead to higher rates of PTSD (170). Efforts have been made to study women in the military, for example, who have been exposed to combat traumas (171). However, many of these women have co-occurring combat and sexual trauma (172), which confounds such studies.

There is also a question of whether biological sex, separate from gender, plays a role in PTSD risk. Heritability of PTSD differs by sex (16), and whether the biological consequences of sex chromosomes, hormones, and reproductive organ physiology contribute to PTSD risk is an outstanding question, separate from gendered factors such as type and degree of trauma exposure.

With regards to sex hormone effects, estrogen and testosterone are steroid hormones with receptors that have intrinsic DNA binding and transcription factor activity (173). Genetic variants in DNA targets of these hormones may impact downstream transcriptional activity in a hormone-dependent manner (174). This may confer differential stress-reactivity based on hormone availability. It is important to note that in animal models or humans both testosterone and estrogen are present at different concentrations throughout development, pregnancy, and the menstrual cycle (175). Therefore, when assuming a hormonal hypothesis of sex effects, it is crucial to measure hormone concentrations rather than using chromosomal or morphological sex as a proxy for hormonal status.

The chromosomal impact on the genetics of stress response is another interesting area of exploration. Variants conferring stress-interactive risk may fall on the X or Y chromosome and mediate chromosomal sex-specific effects (176, 177). With the emergence of CRISPR/-Cas9-based strategies that can eliminate entire chromosomes (178), strategies for matched-donor in vitro modeling of sex chromosome effects have opened up. For instance, cells from donors with Klinefelter’s syndrome with XXY karyotypes may be used, where elimination of either an X or Y chromosome may allow for isogenic experiments assessing the impact of chromosomal availability. Then, applying stressors to these cells may identify chromosomal impacts of stress response, further elucidating chromosomal sex-based mechanisms of stress susceptibility.

In addition to gendered discrepancies in PTSD, PTSD rates are disproportionate by race. In the United States, Native American and Black individuals are disproportionately likely to experience PTSD (179, 180), likely due to high rates of interpersonal traumas present in Black and Native American communities. In particular, Native American individuals report higher rates of combat trauma, rape, physical assault, and childhood sexual abuse (181). Black individuals report higher rates of child maltreatment, domestic violence, and physical assault. In addition to trauma exposure, patients of color additionally experience unbalanced access to healthcare after trauma exposure (182) as well as unequal treatment within the healthcare system itself (183). Black patients, on average, take longer to access care (184), and this is linked to worse outcomes.

When examining the genetics of PTSD, however, racial experiences of disproportionate trauma burden and PTSD susceptibility can confound genetic studies (106). For instance, given the elevated rates of trauma suffered by Black individuals, and that elevated trauma burden heightens risk of PTSD, Black individuals may develop PTSD more frequently —– and crucially, when considering the impact on genetic studies, may require a lower overall genetic burden to develop PTSD compared with groups with lower trauma exposures. On the other hand, White individuals are at substantially lower risk of trauma exposure and thus may require higher genetic risk burdens in order to develop the disorder. These phenotypic disparities may further compound with ancestry-specific differences in allele frequency spectra, further confounding genetic studies. We note that race and ancestry are poor proxies; however, the majority of individuals racialized as Black in the United States have varying degrees of African ancestry.

This highlights the importance of considering trauma in genetic studies of PTSD, as normalizing accounting for the higher rates of racial trauma in certain populations will help identify variants with true associations with PTSD rather than trauma burden.

## Conclusions

While 1 in 5 individuals are currently suffering from psychiatric disorders (185), up to 60% of these individuals will not respond to one-size-fits-all treatment (186). This heterogeneity has prompted investigations into molecular subtypes and endophenotypes underlying various forms of psychiatric disorders, a shift from candidate gene studies aimed at identifying unifying biological explanations for disorder manifestation. Especially as genomic sequencing and molecular profiling expand, the varied impacts of genotype (19, 26, 187–189), fetal exposures (190, 191), adverse childhood experiences (192), medication use (193, 194), and beyond (195) demonstrate the myriad biological pathways implicated in disorder risk. It is likely that individualized approaches will be required to address unique treatment-susceptibility profiles (196), placing emphasis on the development of tools to derive individualized risk from genetic and environmental data.

Strategies to mitigate gender-based violence and gender-based traumas, especially targeted against girls in childhood, are necessary to study and implement to prevent the highly gendered risk of PTSD. Additionally, studies assessing sex hormone effects should consider in vitro exposure of these hormones in addition to physiological mediators of stress, such as glucocorticoids, to observe how hormone availability may impact stress response. It is possible that stress-interactive eQTLs fall on the X or Y chromosomes, and future eQTL studies aimed at considering sex effects should include sex chromosomes in discovering eQTLs. These can then be used to construct TI models of sex chromosomes, which will allow for the imputation of baseline and stress-interactive genetic regulation of expression across multiple disorders.

It is also hugely important to assess highly admixed and genetically diverse cohorts, in order to discover risk variants present across the population. In doing so, it is important to understand the population stratification of traumas, owing to racialized identities and impacts of generational trauma. Future studies should carefully consider the intersection between an individual’s genetic ancestry and their racialization in society, incorporating relevant trauma information to understand whether these individuals experience PTSD due to their experiences of trauma or aspects of their genetic risk.

This shift into deciphering personalized risk has been aided by technological advancements in computational processing power, which has enabled an explosion of new methods in psychiatric genetics. With GWAS now expanded to include millions of individuals (197), genomic discovery is occurring at an unprecedented scale. Matched EHR data offers large-scale clinical insights that can be mapped to genomic risk and biomarkers (198). Advances in natural language processing allow for parsing of clinical notes to generate robust clinical informatics (78). The implications of this include being able to both detect stress and predict risk for stress-related disorders. The explosion of data allows not only for examination of an individual’s particular genetic risk, but also for the matched clinical information to examine how environmental factors may interact with individual genetics at scale, allowing for context-specific investigation.

## Version 1 03/03/2025

Electronic publication
